# Retraction: Theoretical investigations on the effect of absorbent type on carbon dioxide capture in hollow-fiber membrane contactors

**DOI:** 10.1371/journal.pone.0350767

**Published:** 2026-06-03

**Authors:** The PLOS One Editors

The *PLOS One* Editors retract this article [1] due to concerns about compromised peer review.

SS did not agree with the retraction. ATN, AH, and MG either did not respond directly or could not be reached.
